# Developmental Roles of the Hog1 Protein Phosphatases of the Maize Pathogen *Cochliobolus heterostrophus*

**DOI:** 10.3390/jof7020083

**Published:** 2021-01-26

**Authors:** Rina Zuchman, Roni Koren, Benjamin A. Horwitz

**Affiliations:** 1 Faculty of Biology, Technion–Israel Institute of Technology, Haifa 3200003, Israel; rinaz@technion.ac.il (R.Z.); ronik@technion.ac.il (R.K.); 2 Smoler Protein Center, Technion–Israel Institute of Technology, Haifa 3200003, Israel

**Keywords:** protein phosphatases, plant pathogen, plant phenolics, ferulic acid, plant defense, signaling pathways, mitogen activated protein kinase (MAPK), stress response

## Abstract

Protein phosphorylation cascades are universal in cell signaling. While kinome diversity allows specific phosphorylation events, relatively few phosphatases dephosphorylate key signaling proteins. Fungal mitogen activated protein kinases (MAPK), in contrast to their mammalian counterparts, often show detectable basal phosphorylation levels. Dephosphorylation, therefore, could act as a signal. In *Cochliobolus heterostrophus*, the Dothideomycete causing Southern corn leaf blight, ferulic acid (FA)—an abundant phenolic found in plant host cell walls—acts as a signal to rapidly dephosphorylate the stress-activated MAP kinase Hog1 (High Osmolarity Glycerol 1). In order to identify the protein phosphatases responsible, we constructed mutants in Hog1 phosphatases predicted from the genome by homology to yeast and other species. We found that *Cochliobolus heterostrophus* mutants lacking PtcB, a member of the PP2C family, exhibited altered growth, sporulation, and attenuated dephosphorylation in response to FA. The loss of the dual-specificity phosphatase CDC14 led to slow growth, decreased virulence, and attenuated dephosphorylation. Mutants in two predicted tyrosine phosphatase genes PTP1 and PTP2 showed normal development and virulence. Our results suggest that a network of phosphatases modulate Hog1’s dual phosphorylation levels. The mutants we constructed in this work provide a starting point to further unravel the signaling hierarchy by which exposure to FA leads to stress responses in the pathogen.

## 1. Introduction

Protein phosphorylation cascades are universal in cell signaling. Signaling protein activity depends on a balance between protein phosphorylation and dephosphorylation. The vast array of kinases allows for a high degree of specificity in cellular phosphorylation events. In contrast, a relatively small number of phosphatases are responsible for the dephosphorylation of key signaling proteins. For example, the genome of the opportunistic fungal pathogen *Aspergillus fumigatus* encodes 110 kinases (Kinomer database–http://www.compbio.dundee.ac.uk/kinomer/bin/kinomes.pl searched September 2020), while 32 phosphatase catalytic subunits have been identified [1]. In most eukaryotes, kinases comprise as much as 2% of the genome, and are responsible for the phosphorylation of more than 30% of the cellular proteins [2]. The specificity of phosphorylation is derived mainly from the high diversity of the kinome. Phosphatase activity is important for the avoidance of the over-activation of biological processes, and the spatial and temporal restriction of kinase activation [3]. The specificity of phosphatase activity depends, among other properties of the enzyme and substrates, on the amino acid to be dephosphorylated (e.g., Ser/Thr or Tyr; S/T or Y) and on the additional proteins mediating the phosphorylation/dephosphorylation reactions [4,5].

The early work on the response of yeast to hyperosmotic stress characterized the high osmolarity/stress activated MAPK (Mitogen Activated Protein Kinase) Hog1 (High Osmolarity Glycerol 1). Upon the exposure of the cell to high osmolarity, Hog1 undergoes hyperphosphorylation, promoting its translocation to the nucleus. In the nucleus, Hog1 stimulates the expression of a set of genes involved in glycerol production and uptake, and thus balances the osmotic differences and prevents cell death [6]. Most fungal genomes encode 3–4 MAP kinases, which are central to development and virulence of pathogenic species [7]. Hog1 orthologs of filamentous fungi play roles not only in stress tolerance but also in development and virulence.

The exposure of the maize pathogen *Cochliobolus heterostrophus* to ferulic acid (FA) results in the rapid dephosphorylation of Hog1 [8]. FA, a ubiquitous plant phenolic present in the cell walls of stems and leaves, is thought to act as an antimicrobial defense molecule. It is covalently linked to lignin and proteins by ether bonds, and to polysaccharides by ester bonds, acting as a universal connector between cell wall polymers. Upon a plant–pathogen encounter, FA is released by fungal esterases. FA plays an important role in strengthening the cell wall by anchoring lignin to the wall polysaccharides, thus restricting the accessibility of plant pathogens [9,10]. The exposure of the fungus to FA causes hyphal shrinkage, membrane damage, and regulated cell death [8,11,12]. Previous work revealed that FA acts as a stressor, causing rapid cell death with some hallmarks of programmed/regulated cell death (PCD/RCD) [7,12]. Quite unexpectedly, exposure to FA strongly suppressed dual phosphorylation at the TXY motif of the MAPKs Chk1/Pmk1 and Hog1 [8]. 

Usually, dual threonine and tyrosine phosphorylation (at the TXY motif) activates MAPKs in response to signals. We propose two hypotheses to explain why FA exposure dephosphorylates Hog1, rather than raising the dual-phosphorylation level. (1) Lowering Hog1 phosphorylation mitigates a cell-death–promoting signal. In support of this, data from a yeast link sustained Hog1 phosphorylation to cell death [13,14]. Likewise, the fungicide fludioxonil causes the sustained activation of Hog1, killing fungal cells [15]. Furthermore, the deletion of Hog1 increased the sensitivity of *C. heterostrophus* to FA toxicity [8]. (2) The dephosphorylation of Hog1 could, in itself, transduce a host-derived signal provided by FA. In support of hypothesis (2), exposure to FA extensively reprograms the fungal transcriptome [16]. These two hypotheses are not mutually exclusive. Both the lack of active Hog1 and its over-activation could compromise survival. If so, the homeostasis of the Hog1 dual-phosphorylation levels would be essential for tolerance toward FA. In order to investigate these two mechanisms and the role of dephosphorylation, we identified phosphatase genes.

The genome of *C. heterostrophus*, the agent of Southern corn leaf blight, was among the first Dothideomycetes sequenced [11,17]. In fungi, several phosphatases have been proposed as Hog1 modulators: PtcB, a S/T phosphatase of the PP2C family [1]; CDC14, a cell-division–related phosphatase with dual specificity; and PTP1 and PTP2, tyrosine phosphatases [18,19,20]. PtcB was identified as a high osmolarity glycerol response phosphatase in *Aspergillus fumigatus*; a ΔPtcB strain showed increased SakA (Hog1 ortholog) and MpkA phosphorylation, the higher expression of osmostress-dependent genes, higher sensitivity to cell-wall–damaging agents, and impaired biofilm formation [1]. PtcB orthologs regulate Hog1’s phosphorylation state in vivo, both at the basal level and during adaptation to stress, via dephosphorylation at Thr-171, which is located in the activation loop along with Tyr-173 [19,21].

CDC14 is studied in the context of cell division, but its role in multi-stress responses has generally been overlooked, although it could interact with other phosphatases and MAPKs [22,23]. There is evidence that CDC14 and Hog1 are somehow linked; for instance, Hog1 phosphorylation is reduced in *Beauveria bassiana* mutants lacking CDC14 [24], and a theoretical study modelling the cell cycle effects of osmotic stress in yeast predicted that the two are indirectly connected [25]. Despite these hints, Hog1 was not recovered among the physical interactors of CDC14 in a proteomic study on *Candida albicans* [26], suggesting that further work is required in order to clarify the nature of the interaction or link between these two proteins.

PTP2 overexpression in *Magnaporthe oryzae* counteracted the action of fludioxonil [15]. This suggests that PTP2 orthologs might serve as endogenous regulators of Hog1. Indeed, in *Cryptococcus neoformans,* Ptp2 suppressed Hog1 hyperphosphorylation, and its activity was required for wild-type vegetative growth, sexual differentiation, stress responses, antifungal drug resistance, and virulence factor regulation. Ptp2 apparently acts in *C. neoformans* through the negative-feedback loop of the HOG pathway. Interestingly, the absence of PTP2 did not compromise its viability, since PTP1 served as a complementary PTP to control some of the stress responses [27]. Collectively, the findings from the diverse fungal species detailed above suggest that the four candidate genes—PtcB, CDC14, Ptp1 and Ptp2—could serve as Hog1 modulators in *C. heterostrophus*. Here, we set out to examine this possibility by exploring the effects of FA on the Hog1 pathway, characterizing, by gene deletion experiments, the role of the aforementioned genes in the dephosphorylation of Hog1.

## 2. Materials and Methods

### 2.1. Candidate Protein Phosphatase Gene Search

Amino acid sequences of AfPtcB (*A. fumigatus)*, ScCDC14 (*Saccharomyces cerevisiae)*, ScPTP1, and MoPTP2 (*M. oryzae*) protein phosphatase genes [15,18,19] were retrieved from the Uniprot Database (http://www.Uniprot.org/). Reciprocal BLASTP searches of the JGI *C. heterostrophus* C4 (Ch) database led to the predicted orthologs in *C. heterostrophus*. Phylogenetic trees were constructed with the Phylogeny.fr pipeline–MUSCLE (alignment), GBLOCKS (gap curation), PhyML (phylogeny) and TreeDyn (tree plotting) at http://www.phylogeny.fr/ [28]. The phylogenetic trees included the predicted *C. heterostrophus* candidate orthologous genes from alignments with different fungi such as *A. fumigatus (Af)*, *S. cerevisiae (Sc)*, *M. oryzae (Mo)*, *C. neoformans (Cf)*, *Alternaria alternata (Aa)* and other organisms representing outgroups.

### 2.2. DNA Manipulations and Construction of C. heterostrophus Transformation Vectors

Split marker PCR products (~10–15 g total) were designed (primer sequences are given in Table 1), in order to replace the genes of interest (see Table 2: PtcB, CDC14, PTP1 and PTP2) with a hygromycin resistance cassette as described by Catlett at al. [29]. DNA fragments were amplified and cloned using the pGEM^®^-T Easy Vector Systems (Promega, Madison, WI, USA) according to the manufacturer’s instructions. Plasmids were transformed into *E. coli* DH5α and extracted using A Mini-Prep DNA extraction kit (Macherey-Nagel, Düren, Germany). The plasmids were linearized using restriction enzymes, and introduced into the fungi by protoplast transformation (as detailed below).

### 2.3. Generation and Transformation of Fungal Protoplasts.

Fungal protoplasts were generated and transformed as described in Turgeon et al. [30]. In brief, *C. heterostrophus,* race T strain C4, was grown in 90 mm Petri dishes with (complete medium, xylose, CMX), at 22 °C for about 1 week, in a 12 h light/12 h dark regime. A conidial suspension from these plates was germinated overnight, as follows: the spores were inoculated to 150 mL (complete medium, CM), in a rotary shaker for 18 h at 30 °C, 200 rpm, and used for the preparation of the protoplasts. The fungal suspension was centrifuged at 8000 rpm for 10 min (Sorvall, Thermo Fisher Scientific, Waltham, MA, USA; SLA1500 rotor). The resulting mycelium sediment was suspended with 70 mL enzyme-osmoticum solution (see [30]; β-glucanase was replaced by an equivalent amount, following calibration, of VinoTaste enzyme preparation, Novozymes A/S, Bagsvaerd, Denmark) and divided into five 125 mL Erlenmeyer flasks, followed by incubation for 2.5 h at 30 °C, 70 rpm, in a rotary shaker. The protoplasts released were filtered through gauze. The filtrate was then centrifuged at 4500 rpm for 5 min at 4 °C. The protoplast pellets were washed with 10 mL 0.7 M NaCl, followed by a second wash in 10 mL sorbitol–Tris–calcium (reagents were from Sigma, Saint Louis, MO, USA, unless noted otherwise) buffer (STC, see [30]).

The resulting protoplasts were suspended in 200 µL of STC and counted. A concentration of 10^8^ cells/mL was obtained, and of these, 10^7^ protoplasts were used for the transformation. The protoplasts were incubated on ice with 25 µg DNA for 12 min (a negative control without DNA was subjected to the same procedure). Polyethylene glycol was added in three aliquots of 200, 200, and 800 µL each, and then diluted with 1 mL STC and plated for regeneration. A 1% agar overlay containing 100 µg/mL Hygromycin B (HYG, catalog number H0654, Sigma, Saint Louis, MO, USA) was added after 18 h, for selection. For the protoplast viability determination, one of the control plates was overlayed with medium without HYG.

### 2.4. Single-Spore Isolation

Newly-formed colonies after transformation were transferred to CMX–HYG 100 µg/mL plates. Once formed, the spores were collected and diluted in sterile water so that up to 10 spores were dispersed on a 25 mL CMX–HYG 100 µg/mL plate, resulting in the formation of new colonies, each originating from a single spore.

### 2.5. Transformant Verification

The gene replacement by the Hygromycin resistance cassette (HYG) was verified by PCR. Amplification with primers designed from the gene sequence (ORF FR and ORF RV, Table 1) confirmed the loss of the ORF, to levels below detection. Amplification with a primers upstream of the 5′ gene flank used to construct the knockout mutant (HI Upstream, Table 1) paired with a primer within the hygromycin phosphotransferase cassette (NLC37 and NLC38, Table 1) confirmed the replacement of the desired gene by the HYG resistance marker.

### 2.6. Growth Rate Measurements and Phenotypes

*C. heterostrophus* strains were grown in CM agar plates with or without 1 M sorbitol, and on complete medium with dimethylsulfoxide (CM-DMSO) agar plates with or without 2 mM FA (DMSO is the solvent for FA stock; the final DMSO concentration 0.15% *v/v*), for up to 14 days (8 h/16 h dark/light cycles). The radial hyphal growth was measured every 1–2 days, and the colony morphology was imaged using an Olympus (Olympus Life Science, Waltham, MA, USA) SZX16 fluorescence binocular microscope. The spores were imaged using a Leica (Leica Camera AG, Wetzlar, Germany) DMI8 inverted microscope, and the colonies were imaged using a cellphone camera. After 14 days, conidia from an area of 4 cm^2^ were collected in 0.8 mL liquid CM and filtered through double layered gauze in order to clear residual hyphae from the spore suspension. In total, 5 µL were allowed to set on a cover slip in a humid chamber for 1.5 h. Pictures were taken using a Leica DMI8 inverted microscope. For the adhesion assays, each droplet was washed twice with 50 µL pure deionized water (DDW) and documented again. The image processing was performed using an LAS X program (https://www.leica-microsystems.com/products/microscope-software/p/leica-las-x-ls/).

### 2.7. Hog1’s Phosphorylation State in Response to Different Stimuli

*C. heterostrophus* strains (8 cubes of about 8 mm^3^, taken from the margins of wild type (WT), dPTP1 and dPTP2, and 20 cubes of dPtcB and dCDC14 colonies) were each grown in 200 mL CM in a shake culture (24 °C, 185 rpm) for 40 h. Each was then divided equally into four Erlenmeyer flasks, followed by the addition of either sorbitol or FA (1 M and 2 mM final concentrations, respectively). For the sorbitol and FA, liquid CM and CM supplemented with the solvent, and DMSO at matching concentration (0.15% *v/v*) served as controls, respectively. The fungus was further incubated for 5 and 30 min, respectively, and then separated from the liquid medium by filtration on gauze, and immediately frozen in liquid nitrogen.

### 2.8. Immunoblotting

Frozen mycelia from *C. heterostrophus* strains were ground in liquid nitrogen, and their proteins were extracted using SDS buffer containing 5% SDS, 100 mM Tris pH 8, and 10 mM DTT, heated for 5 min at 95 °C and sonicated (30 s, level 5, Microson XL-Misonic, Farmingdale, NY, USA). The extract was then centrifuged (10,000 g, 10 min, room temperature), and the supernatant was subjected to acetone precipitation. The resolved protein pellet was solubilized using a urea buffer containing 8 M urea, 100 mM ammonium bicarbonate and 10 mM DTT. The protein concentration was determined using the Bradford assay, and 25 µg from each extract was loaded onto a 10% SDS-PAGE. The transfer was performed using the e-blot protein transfer kit (GenScript, Piscataway, NJ, USA). The blots were exposed to either p-P38 antibody (Cell Signaling, Danvers, MA, USA, T180-Y182, 9211S) or Hog1 antibody (Santa Cruz Biotechnology, Dallas, TX, USA, y-215, sc-9079) in order to examine the changes in the Hog1 phosphorylation levels or the total Hog1 levels, respectively, upon exposure to different treatments (sorbitol or FA). A luminescence detection by ECL was performed in a Fusion Pulse (VILBER Lourmat, Collégien, France) imager.

### 2.9. Virulence Assays

Seedlings of sweet corn (local hybrid, Royalty) were grown for about 3 weeks, until the fourth leaf began to expand. Next, 1 mm^3^ cubes of *C. heterostrophus* strains were attached to the leaf using clear tape in a moist environment (a closed plastic bag) and allowed to set for 24 h, after which the tape was removed and the plants were grown for four more days. The lesion areas were measured using ImageJ (public software, National Institutes of Health, Bethesda, MD, USA).

### 2.10. Complementation Analysis

In order to rule out the possibility that the phenotypes detected in the PtcB deletion mutants resulted from other mutations, a complementation analysis (AddBack [AB]) was performed. The PtcB genomic sequence was amplified from C. *heterostrophus* C4 using a PCR reaction performed with FP1 and RP2 (Table 1). The complementation was performed by co-transformation, with the full PtcB gene and plasmid pNG conferring neomycin resistance. The colonies growing through the overlay with 1 mg/mL neomycin underwent single sporing, as described above. The transformants were verified by PCR, first by amplification with primers designed from the gene sequence (ORF) verifying the insertion of the gene, and subsequently with primers from the hygromycin phosphotransferase cassette, in order to determine whether the insertion of the gene replaced the hygromycin resistance cassette at the PtcB locus. The phenotypes were tested for three AB transformants that were positive for the ORF.

## 3. Results

### 3.1. Identification of Protein Phosphatase Genes

**Figure 1:**
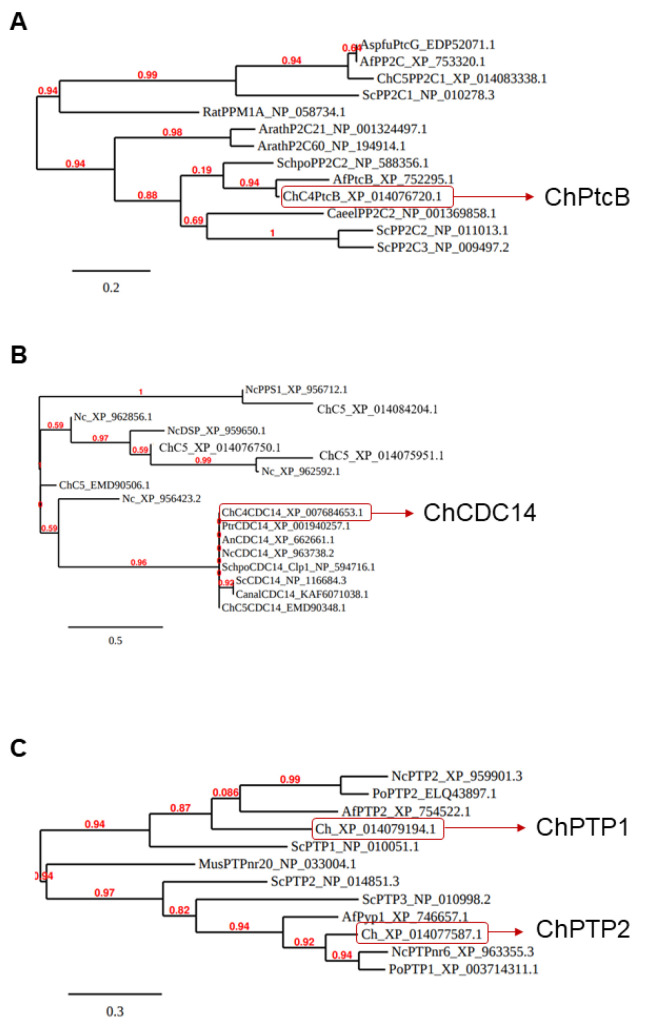
*C. heterostrophus* candidate phosphatase-encoding genes. (**A**–**C**) Following reciprocal BLASTP searches, the phylogenetic trees of the predicted *C. heterostrophus* phosphatase candidate ortholog genes were generated using the Phylogeny.fr pipeline (for details, see Methods and the legend to Table 2). Each entry in the trees starts with an informative gene name (organism: Nc, *Neurospora crassa*; Ch, *Cochliobolus heterostrophus*; Mus, mouse, Cael, *C. elegans*) followed by an abbreviated phosphatase class name and, last, the NCBI protein database ID.

**Figure 2:**
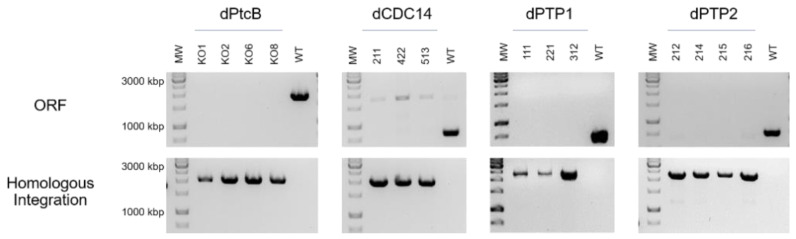
PCR verification of the gene replacement. ORF: amplification of an internal part of the ORF of each gene was performed using primers ORF-FR and ORF-RV. Homologous Integration: a 5′ region of the integrated HYG gene with the upstream flank was amplified, using primers HI–Upstream and NLC37 (Table 1) for the indication of the HYG gene insertion. The numbers indicate independent transformants.

The four putative Hog1 modulators presented in the Introduction section, each containing a different structural phosphatase superfamily domain, were searched with BLASTP. Predicted orthologs were identified (Table 2, Figure 1 and Figure S1). *C. heterostrophus* knock-out mutants were then generated in order to examine the effects of the deletion of each of these genes. Four independent transformants of dPtcB, three of dCDC14, three of dPTP1, and four of dPTP2 were generated. The gene deletion by homologous integration was verified by PCR (Figure 2; see Methods section for details). In each case, the desired gene was indeed missing (negative for ORF). The Hygromycin (HYG) resistance cassette, which was inserted as a substitution for the existing gene, and as a selection marker, was indeed inserted in the correct location (positive for homologous integration) (Figure 2). The insertion of a wild type (WT) copy of PtcB restored the normal phenotypes. In these add-back (AB) strains, the insertions were ectopic, as the hygromycin resistance cassette was retained following single-conidia isolation; the transformants acquired neomycin resistance as well as the complete PtcB ORF (Figures S2 and S3). Perhaps due to the severe phenotypes, we could not obtain transformants in the complementation experiments for dCDC14. The indistinguishable phenotypes of three independently-isolated deletion transformant lines, however, rule out, as far as possible, the possibility that the phenotypes we observed resulted from other mutations.

### 3.2. Phenotypic Characterization of the Protein Phosphatase Deletion Mutants

Following the single-conidia isolation, strains that were positive for Hygromycin resistance cassette-homologous integration and negative for the gene of interest were tested for spore phenotypes, growth rates in different types of media, degree of virulence, and the impact of the FA induction on their Hog1 phosphorylation state. These criteria will be described in detail, below, for each of the deletion mutants.

### 3.3. Spore and Colony Morphology

**Figure 3:**
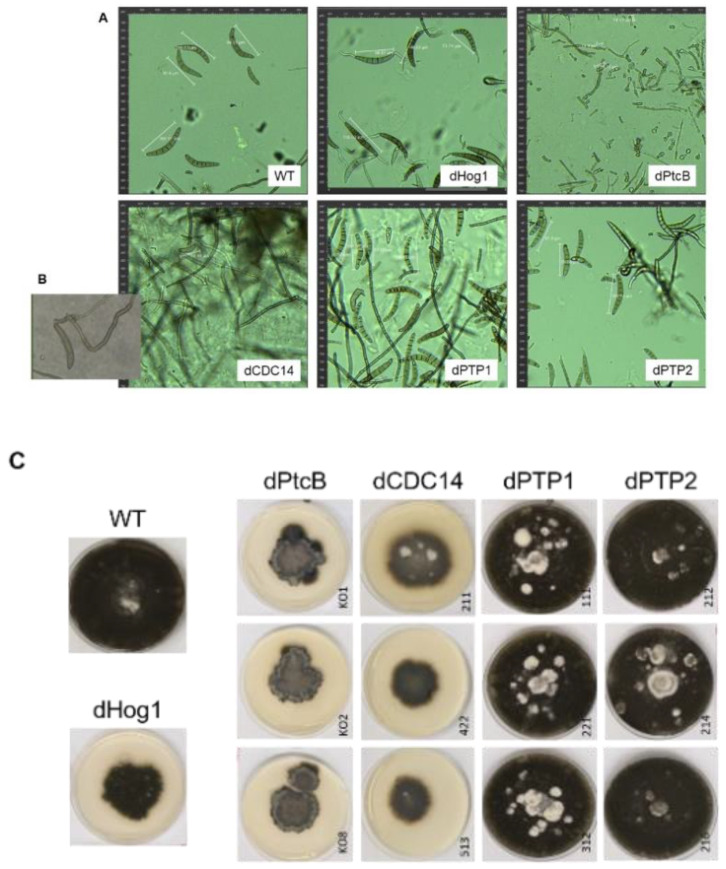
Representative spore (conidia) phenotypes of the WT and mutants. (**A**) Spores were collected and incubated in CM for 1.5 h (see Methods). Pictures were taken using a LEICA DMI8 inverted fluorescent microscope. (**B**) The germination of dCDC14 in a liquid Complete Medium-Xylose (CMX). The conidiation of dCDC14 is sporadic and rare (panel A, lower left), but the spores that were collected can germinate, as shown in this image. (**C**) Colony phenotypes of the WT and mutants. From each mutant, at least three independent transformants were grown on CMX for 12 days, and their pictures were taken.

**Figure 4:**
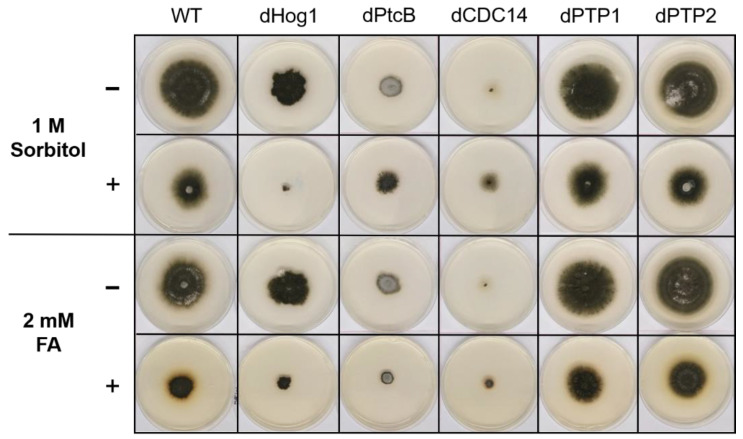
Colony phenotypes under osmotic stress or FA treatment. The cultures were grown for 6 days following their inoculation on CM agar plates with or without sorbitol (− or + 1 M sorbitol), or CM with 0.15% *v/v* DMSO with or without FA (− or + 2 mM FA).

**Figure 5:**
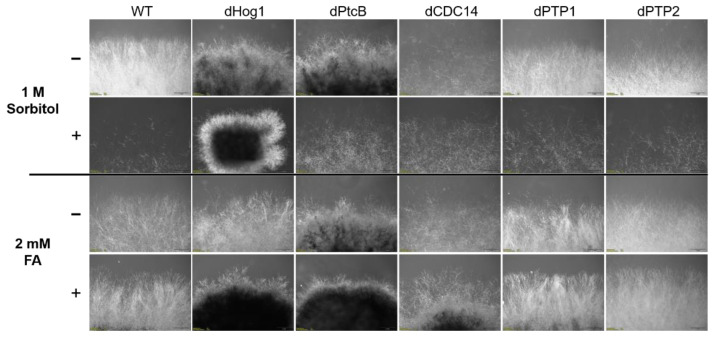
Morphology of the colony margins. The cultures were grown on CM agar plates with or without sorbitol (− or + 1 M sorbitol), or CM with 0.15% *v/v* DMSO with or without FA (− or + 2 mM FA). The pictures were taken 5 days after the inoculum transfer.

**Figure 6:**
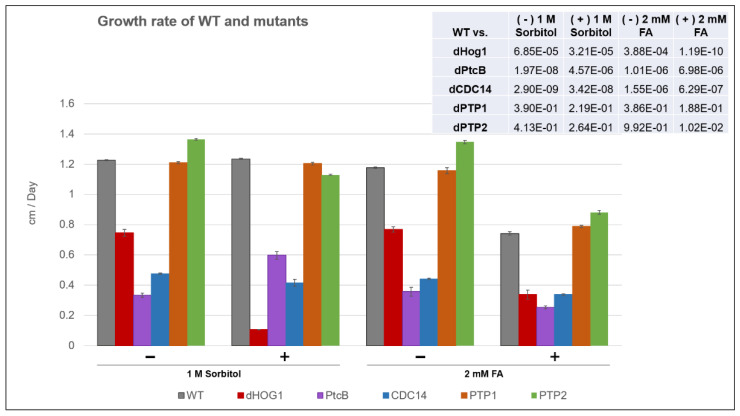
Growth rate of the mutants and WT. The WT and mutants were grown on CM agar plates with or without sorbitol (− or + 1 M sorbitol), or CM with 0.15% *v/v* DMSO, with or without FA (− or + 2 mM FA), for up to 14 d, and the colony radius was measured at intervals (see Methods). The error bars indicate SE. The inset shows the t-test *p* values for the indicated comparisons.

The dPtcB mutants exhibited an abnormal spore phenotype (Figure 3A); this included a shorter spore length (about 20 µm in the mutants compared to 100 µm in the wild type), a lack of cell compartments in the conidia, and a lower spore count in the mutants (Figure 3A). Furthermore, the colonies had a dramatically altered morphology (Figure 3C, Figure 4 and Figure 5), and were slow growing (Figure 6). The dCDC14 mutants almost completely lacked conidia, and the few conidia present lacked the compartmentation into cells that is seen in WT spores. The spore length ranged from about 50 to 70 µm (Figure 3A). Despite the spore structure phenotypes, both the dPtcB and dCDC14 spores were able to germinate (an example is shown for dCDC14 in liquid CMX medium, Figure 3B). The dPTP1 and dPTP2 mutants exhibited wild-type growth, spore cell compartments, and colony morphology (Figure 3A and Figure 4).

The addition of sorbitol to the CM media resulted in longer hyphae compared to CM alone (Figure 5, top panel). From visual inspection, we noted that the hyphae tended to branch to a lesser degree and were generally more invasive, i.e., they grew deeper into the agar (Figure 5, top panel). Although the dHog1 mutants barely grew in the presence of sorbitol, high osmolarity did not promote invasive growth, and the hyphal pattern was essentially normal, with the production of aerial hyphae (Figure 5). The dPtcB mutants showed an altered, disoriented hyphal pattern, and in the presence of sorbitol, the dPtcB mycelia were highly tumbled, with short branches. The dCDC14 hyphae were tumbled and the colony was less dense, yet it seems that high osmolarity encouraged hyphal branching, even in comparison to the WT. In Figure 5, the minus-FA controls show a slightly different morphology from the minus-sorbitol controls. These differences can be attributed to the presence of DMSO, the solvent for FA. Thus, DMSO caused the hyphae to grow in less density and in disorder, although the radial growth rate was not strongly affected (see Figure 6). The addition of ferulic acid (FA) to the CM media led to increased hyphal density, but with almost normal growth patterns (Figure 5, bottom panel). The dHog1 hyphae were shorter in comparison to the WT. The dCDC14 on DMSO grew as on the CM without DMSO, but again, in the presence of FA, the hyphal growth pattern was similar to the WT (Figure 5) despite the slow growth of dCDC14 (Figure 6). When coping with FA, the colony tends to be denser. The reduction of the linear growth could reflect the ability of the fungus to cope with stress. Both dPTP1 and dPTP2 behave as the wild-type when exposed to either sorbitol or FA.

### 3.4. Growth Rate, Germination and Germling Adherence

**Figure 7:**
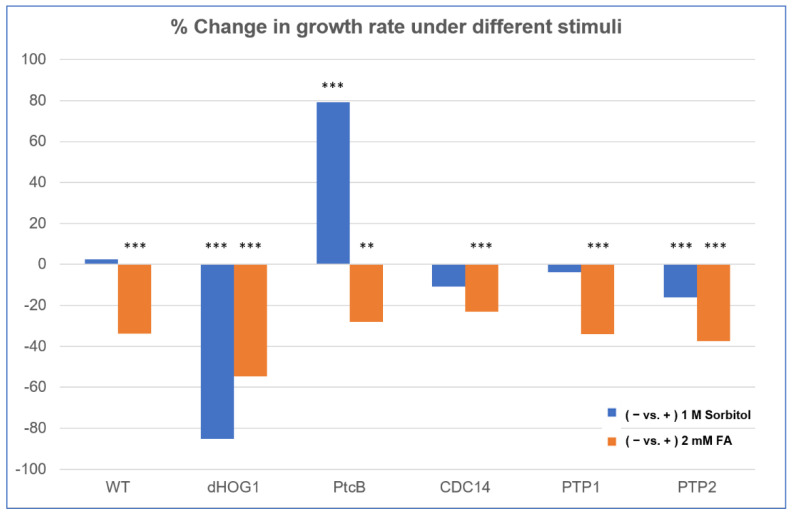
Comparison of FA and sorbitol’s effects on the different strains. The data from Figure 6 are replotted as ratios. CM agar plates with or without sorbitol (− or + 1 M sorbitol) or CM with 0.15% *v/v* DMSO with or without FA (− or + 2 mM FA) were compared. The *t*-test *p*-values are marked as follows: *p*-value < 0.05—**; *p*-value < 0.01—***.

**Figure 8:**
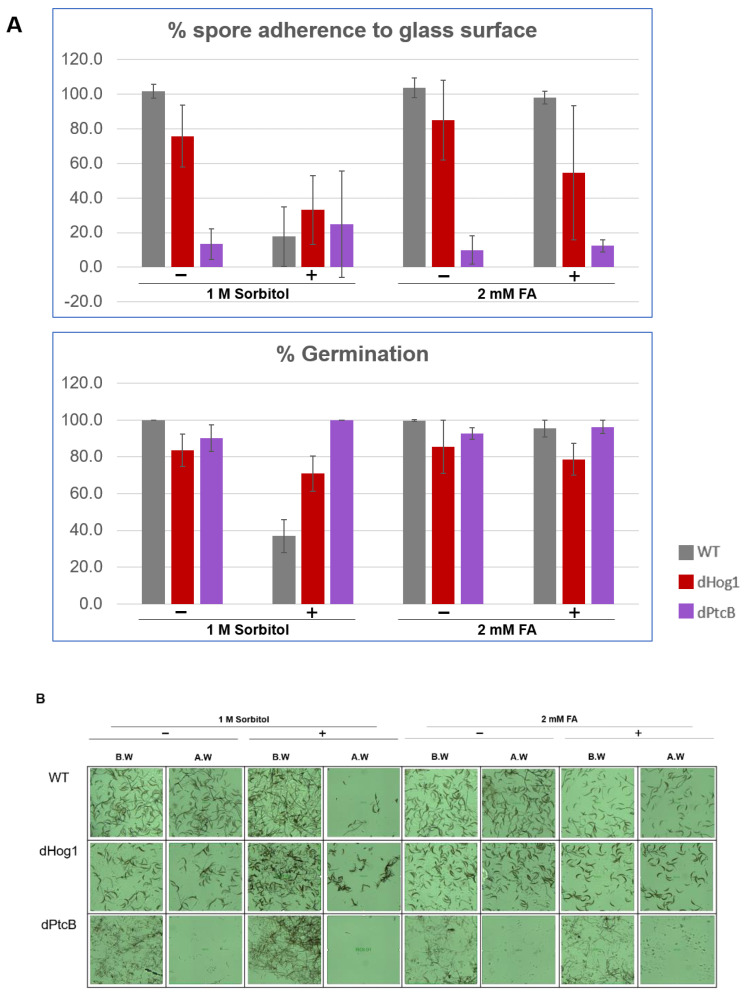
Spore attachment and germination of strains on stress-inducing growth media. (**A**) The percentage of the spore attachment and germination. *C. heterostrophus* C4 WT strain and mutants were grown in CM agar plates with or without sorbitol (− or + 1 M sorbitol), or CM with 0.15% *v/v* DMSO with or without FA (− or + 2 mM FA), for 14 d (8 h/16 h dark/light cycles). Spore suspensions were collected from each, and were incubated on a cover slip in a humid chamber for 1.5 h (see Methods). The pictures were taken using a Leica DMI8 inverted microscope. Each droplet was washed twice with 50 µL DDW and documented again. The image processing was performed using the LASX program. Standard deviation bars were added. (**B**) Representative pictures corresponding to the treatments in panel A.

The linear radial growth of the WT and mutants is shown in Figure 6 (here and in the following figures, the bar graphs follow a color code identifying each strain). The data are replotted as ratios to show the relative effects of sorbitol and FA (Figure 7). It should be noted that, in some cases, the colony perimeter is not easily visible in Figure 5 due to invasive hyphae; the colony perimeters were marked for measurement at the maximum radius of the colony including invasive hyphae within the agar medium. As can be seen from Figure 6 and Figure 7, along with Figure 4, although DMSO is known to affect cell membranes [31], at the low concentration (0.15%) used to apply the FA, it did not affect the radial growth rate of any of the strains tested. Moreover, in the absence of functioning Hog1, high osmolarity strongly decreased the growth rate, as expected [32]. In the presence of FA, the WT growth rate decreases (Figure 7). On the other hand, it was interesting to see that in the WT, spore morphology, attachment, and germination were highly impaired in the presence of high osmolarity, but very much less affected by the presence of FA. Additionally, as expected, dHog1 had difficulty growing at a high osmolarity. Relative to WT, dHog1 grew better in the presence of FA than at a high osmolarity, suggesting different pathways for high osmolarity and FA responses, though both affect Hog1’s dual phosphorylation. The variability in spore germination and attachment was high (Figure 8A), but the general trend may indicate, again, better attachment and germination in the presence of FA than sorbitol. The dPtcB and dCDC14 mutants showed a slower growth rate compared to the WT. Strikingly, dPtcB grew better on CM+sorbitol than on CM. Thus, dPtcB had a relatively better ability to cope with high osmolarity than with the presence of FA (Figure 4B and Figure 6). In this sense, dHog1 and dPtcB have opposite stress sensitivities (Figure 7). The attachment and germination of dPtcB conidia were highly variable and generally impaired (Figure 8). The impaired germination is in agreement with the data for the PtcB ortholog of *A. fumigatus* [1]. The germination and attachment rates could not be measured for CDC14 due its low spore count; however, as noted above, the few spores formed were able to germinate. The deletion of PTP1 did not affect the growth rate, in accordance with previous reports [27]. dPTP2 showed a trend toward a slightly increased growth rate on all media except for CM+sorbitol; the difference was significant only in the presence of FA (Figure 6).

### 3.5. Virulence Assays

**Figure 9:**
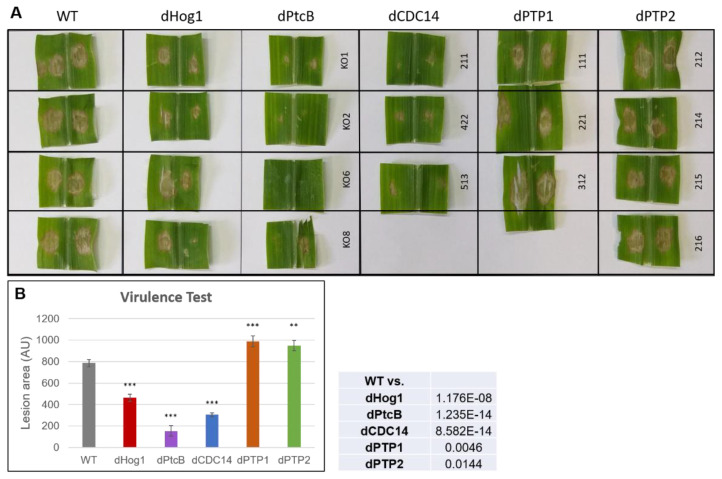
Virulence assay of mutants and WT on maize leaves. (**A**) The third leaf of corn plants (the rounded, juvenile leaf was counted as leaf ‘zero’) was infected with a ~0.09 cm^2^ agar block inoculum for 24 h, and was then removed (see Methods). The lesions were measured 4 days from infection. (**B**) Quantitation of the lesion areas. The lesion areas were measured using ImageJ. The graph represents the average lesion area of each strain, calculated from 3 biological repeats for each independent transformant (for the photos of all of the lesions analyzed, see Supplementary Figure S2). The error bars indicate SE. The *t*-test *p*-values of each strain vs. the WT were calculated and are marked as follows (inset): *p*-value < 0.05—**; *p*-value < 0.01—***.

The deletion of either PtcB or CDC14 strongly suppressed virulence (Figure 9 and Figure S4), as reported for dPtcb in *A. fumigatus* on a mammalian host [1], implying a conserved loss of pathogen fitness. Though the growth rate of both the dPTP1 and dPTP2 mutants on CM was not significantly higher than the WT, these mutants were significantly more virulent than the WT, indicating additional factors in the virulence of the mutants compared to WT, other than simply the effects of slower or faster growth rates.

### 3.6. The Effect of Phosphatase Deletions on Hog1 Dual Phosphorylation

**Figure 10:**
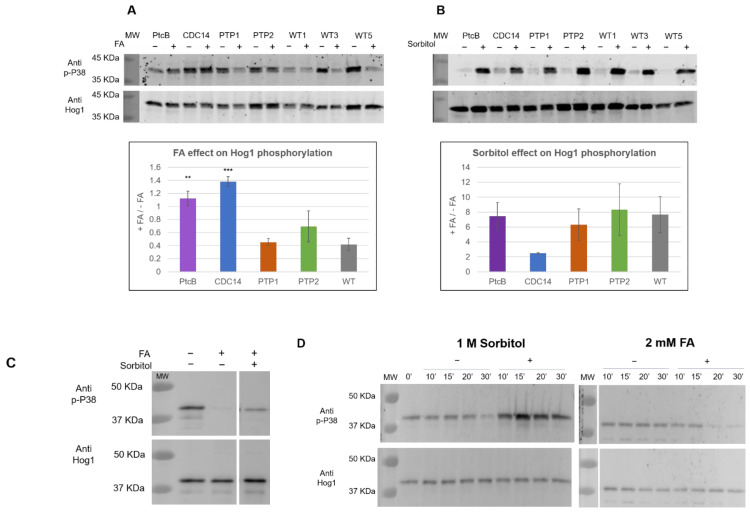
FA and sorbitol’s effects on Hog1 dual phosphorylation in WT and phosphatase mutants, assayed by immunoblotting. (**A**) FA, (−) indicates CM with 0.15% *v/v* DMSO (control); (+) indicates the addition of 2 mM FA. (**B**) Sorbitol, (−) indicates CM; (+) indicates the addition of 1 M sorbitol. Blots of the total protein extract of the WT and the different mutants were probed with either a p-P38 antibody (Cell Signaling, T180-Y182, 9211S) or a Hog1 antibody (Santa Cruz, y-215, sc-9079). Representative blots are shown (for the complete data set, see supplementary Figure S5). The graphs represent the ratio change in the signal upon induction (calculated from three independent deletion transformants, indicated by numbers labelling each lane). The quantitation was performed using Image J. The *t*-test *p*-values of each strain vs. the WT were calculated, and are marked as follows: *p*-value < 0.05—**; *p*-value < 0.01—***. (**C**) The sequential effect of FA and osmotic stress on Hog1 in WT. The samples were analyzed as in panels (**A**,**B**). *C. heterostrophus* C4 WT strain protein extract, from CM with 0.15% *v/v* DMSO (−/−), CM with 0.15% *v/v* DMSO with 2 mM FA (+/−), and CM with 0.15% *v/v* DMSO with 2 mM FA, with the sequential addition of sorbitol to 1 M concentration, as described in the method section (+/+). In this panel, different parts of the same blot are shown side by side at the same scale, and this is indicated by a space between the sections. (**D**) Time course for the sorbitol and FA induction. The media and immunoblotting were as they were in panels (**A**,**B**).

In order to explore the involvement of these genes in the Hog1 phosphorylation levels in response to FA, the mutants and WT were given a brief exposure to FA. The phosphorylation was assayed using immunoblots with an antibody to dual-phosphorylated mammalian P38 (anti p-P38), which has been used extensively to detect dual-phophosphorylated Hog1 (pHog1) in fungi (for example, [33,34]). An antibody against yeast Hog1 (anti Hog1) was used to quantify the total Hog1 protein levels. In the WT, the addition of FA resulted in decreased ppHog1 levels (Figure 10A). This was not the case, however, for two of the mutants; in the strains deleted for PtcB or CDC14, there was little or no reduction in the ppHog1 levels upon exposure to FA (Figure 10A). The addition of sorbitol to the CM media rapidly increased the ppHog1 levels, in the WT and in all four mutants (Figure 10B). Two points emerge from these data: first, the modulation of Hog1 dual phosphorylation by FA and sorbitol are not only opposite (Figure 10A,B) but are also genetically separable, because the loss of PtcB or CDC14 affects the response to FA but not to high osmolarity (sorbitol). The second point is that two distinct phosphatases belonging to different classes are both required for dephosphorylation in response to FA (Figure 10A). This suggests multiple modulators for Hog1, but because each phosphatase mutant showed different properties, the question of the mechanism of Hog1 dephosphorylation upon exposure to FA arises. Next, we tested the effect of sequentially adding FA and sorbitol to the CM media. As shown in Figure 10C, the addition of sorbitol partially counteracted the decrease in the phosphorylation levels due to FA exposure.

This shows that the effects are opposite and additive, though the question of the biochemical mechanism cannot yet be resolved. Finally, we studied the time course of the phosphorylated Hog1’s appearance following its exposure to FA and sorbitol, up to 30 min after its exposure to these compounds. As shown in Figure 10D, pHog1 accumulated gradually over time following the exposure to sorbitol. In contrast, pHog1 was dramatically and rapidly reduced following the exposure to FA. Both inducers act in a time frame that is consistent with cellular signaling cascades, but the time course indicates rapid and efficient dephosphorylation, compared to the somewhat-slower phosphorylating activity of the upstream MAPKK. The low molecular weight bands observed (Figure 10C) probably reflect partial proteolytic degradation products of Hog1 (see Figure 5 in [35]); such bands were not observed in the dHog1 mutants, in line with this hypothesis (Roni Koren, unpublished observations).

## 4. Discussion

### 4.1. Hog1 Dephosphorylation as a Signal

Ferulic acid (FA), an abundant plant phenolic, acts as a stress signal in the maize pathogen *C. heterostrophus*, triggering cell death [8] and regulating the transcript levels of a large number of genes [16]. Our observation that exposure to FA leads to the dephosphorylation of an initial, basal level of TXY dual phosphorylation [8] could have (at least) two functional explanations: (1) dephosphorylation, by lowering the activation level of Hog1, could transduce information, for example by lowering the level of an inhibitory transcription factor; (2) the sustained activation of Hog1 can promote cell death in fungi, so that dephosphorylation could serve as a mechanism to allow the cell to mitigate the cell-death–promoting effect of FA. Supporting this possibility, an earlier study in *Cryptococcus neoformans* serotype A H99 [36] showed the constitutive phosphorylation of Hog1, and dephosphorylation upon exposure to high osmolarity. Both putative functions (1) and (2) of the rapid dephosphorylation of Hog1 are thus of interest, and as a first step toward a mechanistic understanding, we identified protein phosphatase candidates. Mutants were constructed in four candidate genes: PtcB, CDC14, PTP1 and PTP2. The results show that, in addition to modulating the Hog1 phosphorylation level, the loss of phosphatases has profound developmental phenotypes. Viable mutants were obtained for all four genes, and at least three independent transformants were verified and analyzed for all of the phenotypes shown. Furthermore, the introduction of a WT copy rescued the phenotypes of dPtcB. PtcB and the CDC14 mutants show a decreased growth rate, while the tyrosine phosphatases PTP1,2 are, for the most part, dispensable for normal growth and virulence. The loss of PtcB has a more drastic effect on colony morphology and growth than CDC14, but CDC14 has an almost equally drastic reduction in virulence (Figure 4, Figure 5, Figure 6, Figure 7, Figure 8 and Figure 9). While loss-of-function mutations are generally expressed as negative phenotypes, an interesting exception was seen for dPtcB following its exposure to sorbitol (CMS, Figure 4), where dPtcB shows increased, rather than decreased, growth compared to the control. This is further emphasized in Figure 7, where the loss of PtcB has opposite effects on sorbitol or FA-containing media to those seen for the other mutants. This is consistent with PtcB being a major modulator of the Hog1-mediated stress response, in agreement with prior findings in *A. fumigatus* [1]. The opposite effects of sorbitol and FA stresses (Figure 7) suggest that distinct PtcB-dependent biochemical pathways are at work in tuning the level of Hog1 activation. The MAP kinase Hog1, and the stress signals that it transduces, are important in fungal development and virulence. A question thus arises as to why in the case of high osmolarity or exposure to fludioxonil, the fungus ‘fights’ regulated cell death (RCD) by increasing Hog1 phosphorylation levels, while upon exposure to FA, the Hog1 phosphorylation levels instead decrease. The biochemical underpinnings of these opposite responses will be a subject for future work.

### 4.2. Contribution of More than One Phosphatase to FA-Induced Hog1 Dephosphorylation

The loss of either PtcB or CDC14 compromises the full WT regulation of phospho TXY Hog1 levels (Figure 10A,B). There is a trend (though not statistically significant) suggesting that PTP2 might be considered as well: dephosphorylation in response to FA appears to be reduced in this mutant (Figure 10A). Prior work in *Neurospora* and *Aspergillus* [16,18] is in line with our observation that more than a single phosphatase acts on Hog1. PtcB andCDC14 (and perhaps PTP2) participate in the dephosphorylation of phospho-Hog1. One explanation may be sequential activity, in which the action of one phosphatase is needed for a second to act. There is a report that mammalian MAPKs, starting in their non-activated state, are phosphorylated first on tyrosine. Upon induction, the tyrosine-phosphorylation level accumulates to a threshold which, once reached, phosphorylation on threonine is added, and the MAPK—in its dual phosphorylated form—is active [37]. This property of a mammalian MAPK raises the possibility that dephosphorylation could, likewise, be sequential, rather than concerted, with the activity of one phosphatase setting the stage for a second one to act. Mutants lacking either would then show a defect in the dephosphorylation of the TXY motif. This general question can be investigated by time-resolved phosphoprotein MS/MS experiments in mammalian cells, yeast, or filamentous fungi. Another explanation could be the indirect developmental consequences of the lack of a particular phosphatase on the activity of others.

### 4.3. Hog1 Phosphatases of C. heterostrophus in Comparison to Other Fungal Models

The developmental roles for the four phosphatase genes studied here have been reported in other fungi. Fungal signaling pathways based on conserved components are nevertheless ‘wired’ differently in diverse species. The PtcB of *A. fumigatus* is involved in attachment and virulence. It also had a remarkable effect on the cell surface, conidia and germling adhesion, biofilm formation, and MpkA phosphorylation, and its involvement in the CWI pathway was also suggested. Furthermore, a dPtcB strain showed impaired virulence in a mouse model for invasive pulmonary aspergillosis [1]. In *S. cerevisiae*, in contrast, Hog1 dephosphorylation is regulated by phosphotyrosine phosphatases (Ptp2 and Ptp3) or phospho S/T phosphatases (Ptc1). This is also in accordance with the Ptc or Ptp-types of phosphatases in the pathogenic basidiomycete yeast, *C. neoformans* [27]. Collectively, the existing literature suggests that PtcB is a central Hog1 phosphatase, and its loss affects a wide variety of cellular processes. Generally, PtcB is discussed in cases of high osmolarity causing high pHog1 levels, yet in our case, PtcB was involved in lowering the pHog1 levels below the basal level. This is a novel role for both PtcB and Hog1.

Early studies in yeast showed that CDC14 plays a key role in regulating late mitotic processes [38]. More recently, however, studies in some filamentous species have revealed that CDC14 is not always an essential gene, and also that it is involved in diverse cellular functions. The deletion of CDC14 in *Metarhizium acridum*, for example, resulted in a defective growth phenotype and multiple nuclei in each hyphal compartment, indicating nuclear division and cytokinesis defects as expected, yet without significant effects on the sensitivity to cell wall damage reagents, or sensitivity to osmotic and oxidative stress [22]. On the other hand, the inactivation of CDC14 in *Beauveria bassiana* resulted in hypersensitivity to oxidative, osmotic, cell wall and mitosis-perturbing stresses [24]. Filamentous fungal orthologs of yeast CDC14 could have previously overlooked functions in multi-stress responses, including the possible interactions of CDC14 with other phosphatases and MAPKs. Our findings in *C. heterostrophus* support this view.

Two protein tyrosine phosphatases (PTPs) were chosen here as candidate Hog1 phosphatases because they are known to modulate the Hog1 phosphorylation levels in several species. The HOG1 phosphorylation levels decreased in *Magnaporthe oryzae* PTP2-overexpressing strains, while PTP2 loss-of function mutants had higher phosphorylation levels of HOG1 compared to the WT [15]. The deletion of PTP2 and PTP3 (these two genes may have redundant functions) affected the sporulation in yeast, where the deletion of these genes blocks cells at an early stage of sporulation before premeiotic DNA synthesis and the induction of meiotic-specific genes [39]. Moreover, PTP2 was shown to be involved in mediating vegetative growth, sexual differentiation, stress responses, anti-fungal drug resistance, and virulence factor regulation in *C. neoformans* through the negative-feedback loop of the Hog1 pathway, while PTP1 was not essential for Hog1 regulation [27]. PTP1 was dispensable despite its Hog1-dependent induction. Interestingly, the loss of PTP2 did not compromise viability, since PTP1 served as a complementary PTP to control some stress responses [24]. In *C. heterostrophus*, the lack of PTP1 and PTP2 in our mutant strains lead to no obvious phenotypes and little, if any, effect on the steady-state basal Hog1 phosphorylation levels or the dephosphorylation of Hog1 in response to FA. This finding again underscores the differences between the species, but should also encourage the construction of double mutants and over-expression strains in future work.

## 5. Conclusions

Hog1 is well-studied in the context of high osmolarity stress, but the FA-induced pathway studied here has revealed new insights regarding the roles of Hog1 in pathogenicity and in developmental pathways. The results indicate a complex relationship between Hog1 dephosphorylation and the response of the fungal cell to chemical stress by plant phenolics, and a multi-phosphatase regulation system of Hog1. Moreover, apparently, the effect of the deletion of a particular tyrosine phosphatase was less critical than a threonine phosphatase. The deletion of PtcB and CDC14 resulted in abnormal phenotypes—including defective spores, a slower growth rate, and decreased virulence—as opposed to the KO of PTP1 and PTP2. These results open many questions regarding the mechanism by which dephosphorylation is sensitized by FA and the nature of the phosphatase(s) involved. We predict that the sensitivity to FA-induced cell death will be altered when FA-induced Hog1 dephosphorylation is compromised. This prediction can be tested with the help of the mutants described here. Understanding the role of protein phosphatases in the FA-Hog1 pathway may be of general significance for the fungal pathogens of plants: the osmotic response pathway is highly conserved, and exposure to plant phenolics is widespread.

## Figures and Tables

**Table 1:** Primer sequences for the construction of *C. heterostrophus* transformation vectors and the verification of mutants. Hph, hygromycin resistance cassette, see Methods.

Gene	JGI Protein ID	Synonym	Sequence (5′ to 3′)	Origin
Hygromycin		M13Rhyg	AGCGGATAACAATTTCACACAGGA	pUCATPH seq. 2865-2888RC—5′ region of hygB
		NLC37	GGATGCCTCCGCTCGAAGTA	pUCATPH seq. 1685-1704—5′ region of hygB
		M13Fhyg	CGCCAGGGTTTTCCCAGTCACGAC	pUCATPH seq. 352-375—3′ region of hygB
		NLC38	CGTTGCAAGACCTGCCTGAA	pUCATPH seq. 2132-2150RC—3′ region of hygB
PtcB	200919	FP1	GGGAGCATGTAGTAAGTGC	forward primer to PtcB 5′ flank
		RP1	TCCTGTGTGAAATTGTTATCCGCTCTTGTCCGGTATCTAGGAGGAC	complementary to M13R—reverse primer to PtcB 5′ flank
		FP2	GTCGTGACTGGGAAAACCCTGGCGCTTGTGCTCACTTGACCTGG	complementary to M13F—forward primer to PtcB 3′ flank
		RP2	GAATCAGTGGCCAGATCCAGTC	reverse primer to PtcB 3′ flank
		ORF FR	GGCCAGACACTCTCCGAGC	
		ORF RV	CTTCTCGCTTGTCTTCTTGGC	
		HI—Upstream	CTCAAGTAGAGGTGAGTATGGG	
CDC14	56964	FP1	GGTGCATGTTCAGGGACGGCC	forward primer to CDC14 5′ flank
		RP1	TCCTGTGTGAAATTGTTATCCGCTGAGCAGGAAGGGGCGACTCTC	complementary to M13R—reverse primer to CDC14 5′ flank
		FP2	GTCGTGACTGGGAAAACCCTGGCGCCACTTCTTAGTCTCCATCTCCATCGC	complementary to M13F—forward primer to CDC14 3′ flank
		RP2	ATCGGCAAAGACCCTGCCCGTC	reverse primer to CDC14 3′ flank
		ORF FR	CGACTTCCTTGCCTTTGCC	
		ORF RV	GTTCTCGTCAATGCGCTGG	
		HI—Upstream	GTGGGGGGAGTTTAGTTGC	
PTP1	142461	FP1	GATGCGATACGATGGTAGGG	forward primer to PTP1 5′ flank
		RP1	TCCTGTGTGAAATTGTTATCCGCTCGCCCTTGAGCTTTGCCTTG	complementary to M13R—reverse primer to PTP1 5′ flank
		FP2	GTCGTGACTGGGAAAACCCTGGCGCTTGGGCGTGAGAACAATGC	complementary to M13F—forward primer to PTP1 3′ flank
		RP2	CCAGACGACAACCGCTAATGAAC	reverse primer to PTP1 3′ flank
		ORF FR	CTGCTAGAGACGTCAATGCC	
		ORF RV	GCACAATCCGCGGGTTGGTAG	
		HI—Upstream	CAAAGGGGACATGGACGCAC	
PTP2	169617	FP1	GGCTTTAGCTTGCGATGGTC	forward primer to PTP2 5′ flank
		RP1	TCCTGTGTGAAATTGTTATCCGCTCGAGACGTGAACGTGGGAAG	complementary to M13R—reverse primer to PTP2 5′ flank
		FP2	GTCGTGACTGGGAAAACCCTGGCGGGCAAAGCTCATTGGGAG	complementary to M13F—forward primer to PTP2 3′ flank
		RP2	CAAGGAAACACAATGCCCCG	reverse primer to PTP2 3′ flank
		ORF FR	CCAACAAACCCAATGCACTCGTC	
		ORF RV	GTAGGGCCATCCGTGCTAG	
		HI—Upstream	CGCCTTTGCTGTCGTCGCC	

**Table 2:** Candidate protein phosphatase gene search.

Family ^a^	Subfamily ^b^	Class/Domain ^c^	Ch Model Name ^d^	JGI Protein ID ^e^	*N. crassa* Homolog Gene ^f^	NCU ^g^
S/T	PPM	PP2Cc	estExt_Genewise1Plus.C_19_t10368	200919	pph-8	04600
PTP	Dual-specificity	DSPc	fgenesh1_pm.3_#_471	56964	cdc-14	03246
PTP1	Classical	PTPc	e_gw1.16.187.1	142461	pty-2	02257
PTP2	Classical	PTPc	estExt_Genewise1.C_12_t30017	169617	pty-3	05364

^a^ Family abbreviations: S/T, serine/threonine; PTP, protein tyrosine phosphatase. ^b^ Subfamily abbreviations: PPM, Mg2+ or Mn2+-dependent protein phosphatase; dual-specificity, serine/threonine and tyrosine phosphatase. ^c^ Class/domain abbreviations: PP2Cc, protein phosphatase 2C catalytic subunit; DSPc, dual-specificity phosphatase catalytic subunit; PTPc, protein tyrosine phosphatase catalytic subunit. ^d,e^ From the JGI *C. heterostrophus* genome project (https://mycocosm.jgi.doe.gov/pages/search-for-genes.jsf?organism=CocheC4_1). ^f,g^ Based on gene tables from Ghosh et al. [18], reciprocal BLASTP searches of the JGI *C. heterostrophus* C4 database, and the construction of phylogenetic trees using the Phylogeny.fr pipeline (MUSCLE (alignment), GBLOCKS (gap curation), PhyML (phylogeny), and TreeDyn (tree plotting) at http://www.phylogeny.fr/ [28].

## Data Availability

All data are available in the manuscript and accompanying supplementary materials.

## Supplementary Materials

The following are available online at https://www.mdpi.com/2309-608X/7/2/83/s1. Figure S1. *C. heterostrophus* candidate phosphatase-encoding genes. (A) Phylogenetic tree of *A. fumigatus* phosphatases (Winkelströter et al. [19], reprinted here with permission via RightsLink). Arrows indicate the choice of representatives of three families. (B–D) Following reciprocal BLASTP searches, phylogenetic trees of the predicted *C. heterostrophus* phosphatase candidate ortholog genes were generated using the Phylogeny.fr pipeline (for details see Methods and the legend to Table 2). Each entry in the trees starts with an informative gene name (organism: Nc, Neurospora crassa; Ch, *C. heterostrophus*; Mus, mouse, Cael, *C. elegans*, followed by an abbreviated phosphatase class name and, last, the NCBI protein database ID). Protein domains indicating functional activity of each phosphatase and its classification appear below each tree. Figure S2. Analysis of PtcB complemented strains (AddBack, AB). (A) PCR verification of gene replacement. ORF- Amplification of the full ORF of the PtcB gene was performed using primers ORF-FR & ORF-RV. Homologous Integration—5’ region of the integrated HYG gene with upstream flank was amplified, using primers HI—Upstream and NLC37 (Table 1) for the detection of the original HYG gene insertion. In some panels different parts of the same gel are shown side by side at the same scale, and this is indicated by a space between sections. (B) WT vs. dPtcB AB colony phenotype. (C) WT vs. PtcB AB growth rate. WT was grown on CMX agar plates. PtcB AB was grown both on CMX agar plates containing HYG (100 µg/mL) and CMX agar plates containing HYG (100 µg/mL) and neomycin (1 mg/mL). (D) Virulence assay of WT vs. dPtcB AB was performed as in Figure 9. (E–F). Effect of FA induction on Hog1 phosphorylation levels in the WT vs. PtcB AB was assayed as in Figure 10A, B. Figure S3. Quantitation of lesion areas from the virulence assays of PtcB AddBack and WT on maize leaves. Methods were as for Figure 9. Figure S4. Quantitation of lesion areas from virulence assay of mutants and WT on maize leaves. Methods were as for Figure 9. Strain M6 (top left) is not related to this study. Figure S5. FA and sorbitol effect on Hog1 dual phosphorylation in WT and phosphatase mutants, assayed by immunoblotting. All biological repeats for Figure 10: blots of total protein extract of WT and the different mutants, with (+) or without (−) sorbitol or FA induction.
