# Supplementary material for: Developmental Roles of the Hog1 Protein Phosphatases of the Maize Pathogen Cochliobolus heterostrophus
Source: J Fungi (Basel). 2021 Jan 26;7(2):83. doi: 10.3390/jof7020083 (PMC7910936; doi:10.3390/jof7020083)
Supplement: Supplementary file 1 [file jof-07-00083-s001.pdf]

Journal of Fungi

MDPI

Supplementary Materials: Developmental Roles of the Hog1 Protein Phosphatases of the Maize Pathogen *Cochliobolus heterostrophus*

Rina Zuchman, Roni Koren and Benjamin A. Horwitz

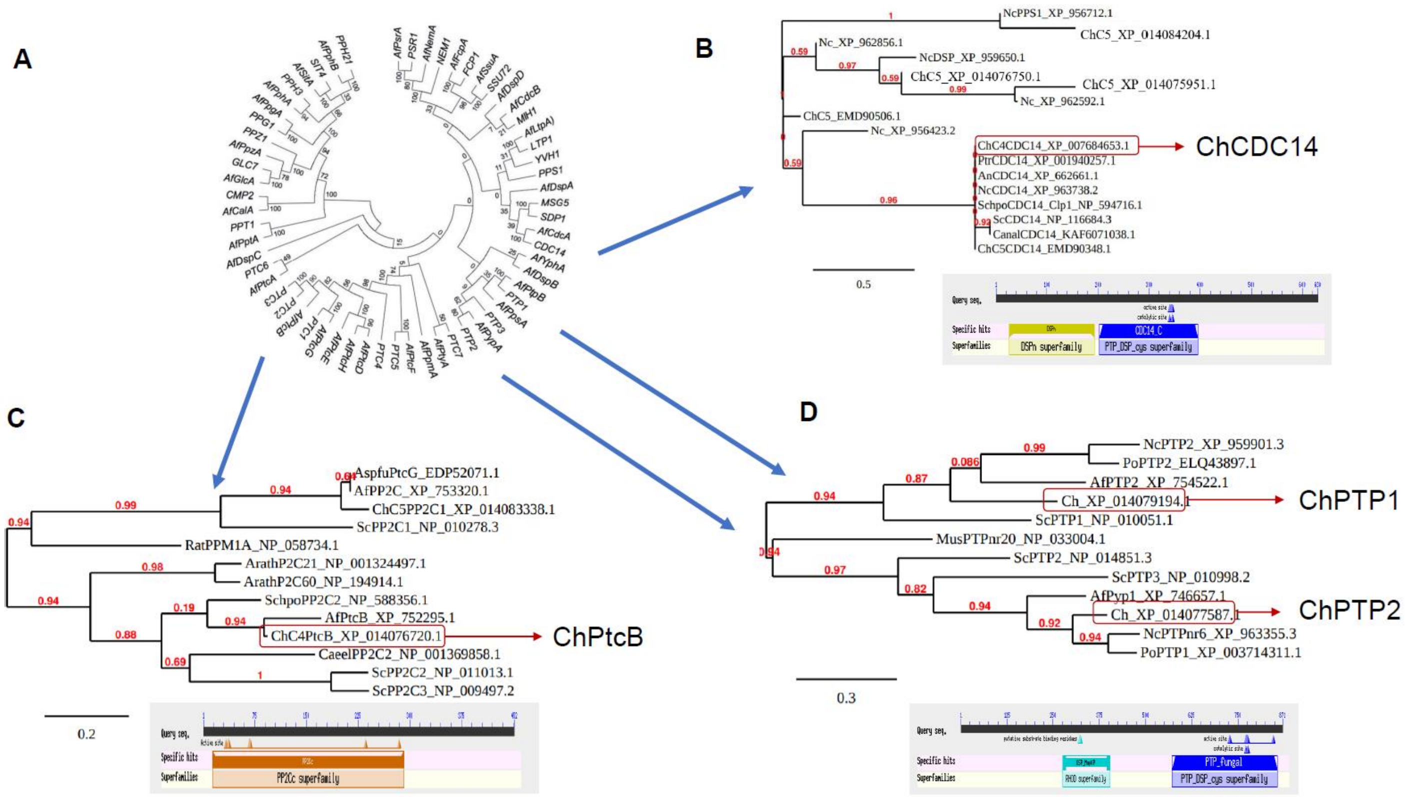

Figure S1. *C. heterostrophus* candidate phosphatase-encoding genes. (**A**) Phylogenetic tree of *A. fumigatus* phosphatases (Winkelströter et al. [19], reprinted here with permission via RightsLink). Arrows indicate the choice of representatives of three families. (**B**–**D**) Following reciprocal BLASTP searches, phylogenetic trees of the predicted *C. heterostrophus* phosphatase candidate ortholog genes were generated using the Phylogeny.fr pipeline (for details see Methods and the legend to Table 2). Each entry in

the trees starts with an informative gene name (organism: Nc, Neurospora crassa; Ch, *C. heterostrophus*; Mus, mouse, Cael, *C. elegans*, followed by an abbreviated phosphatase class name and, last, the NCBI protein database ID). Protein domains indicating functional activity of each phosphatase and its classification appear below each tree.

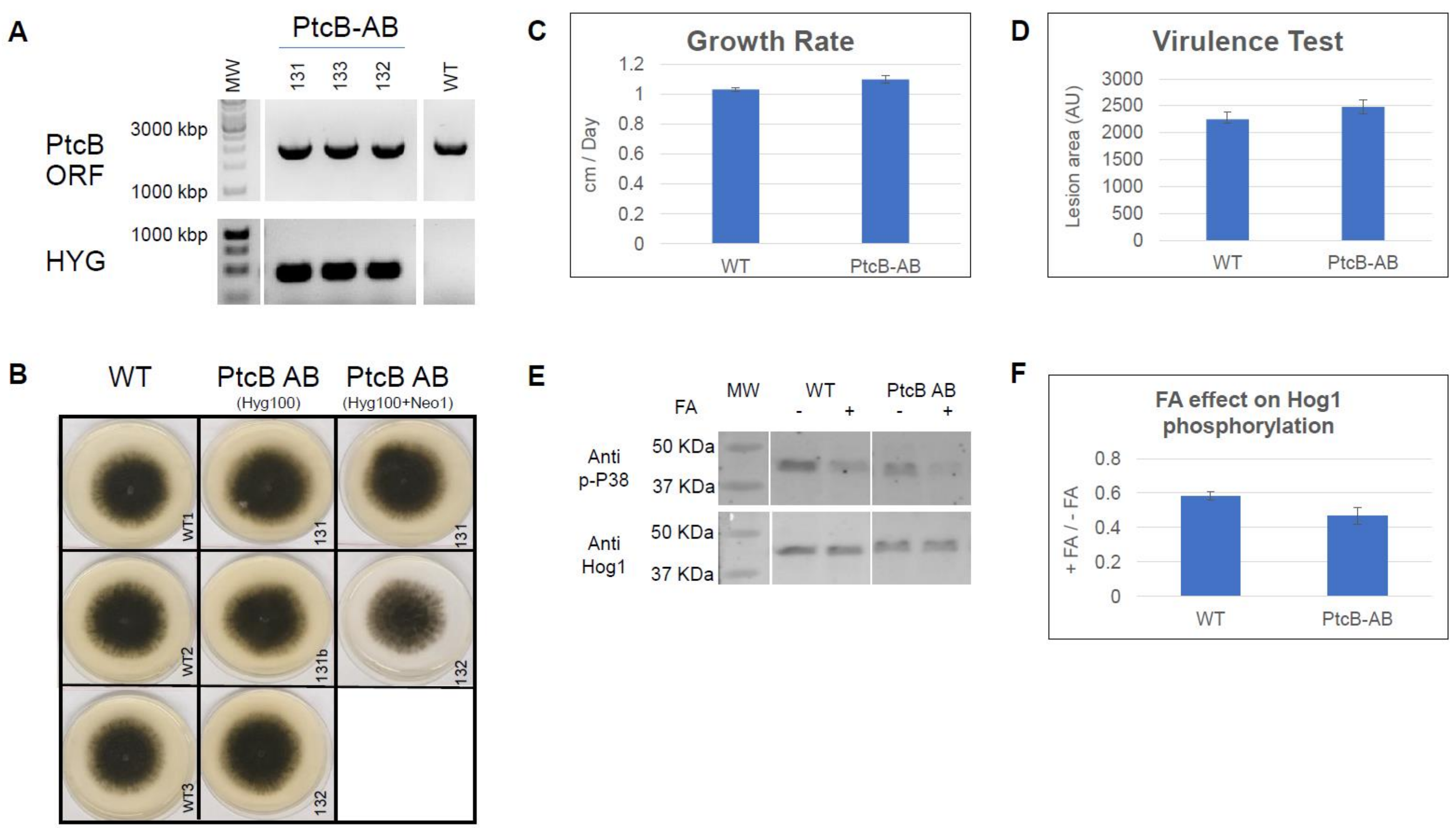

Figure S2. Analysis of PtcB complemented strains (AddBack, AB). (**A**) PCR verification of gene replacement. ORF- Amplification of the full ORF of the PtcB gene was performed using primers ORF-FR & ORF-RV. Homologous Integration - 5′ region of the integrated HYG gene with upstream flank was amplified, using primers HI -

Upstream and NLC37 (Table 1) for the detection of the original HYG gene insertion. In some panels different parts of the same gel are shown side by side at the same scale, and this is indicated by a space between sections. (**B**) WT vs. dPtcB AB colony phenotype. (**C**) WT vs. PtcB AB growth rate. WT was grown on CMX agar plates. PtcB AB was grown both on CMX agar plates containing HYG (100 μg/mL) and CMX agar plates containing HYG (100 μg/mL) and neomycin (1 mg/mL). (**D**) Virulence assay of WT vs. dPtcB AB was performed as in Figure 9. (**E**–**F**). Effect of FA induction on Hog1 phosphorylation levels in the WT vs. PtcB AB was assayed as in Figure 10A, B.

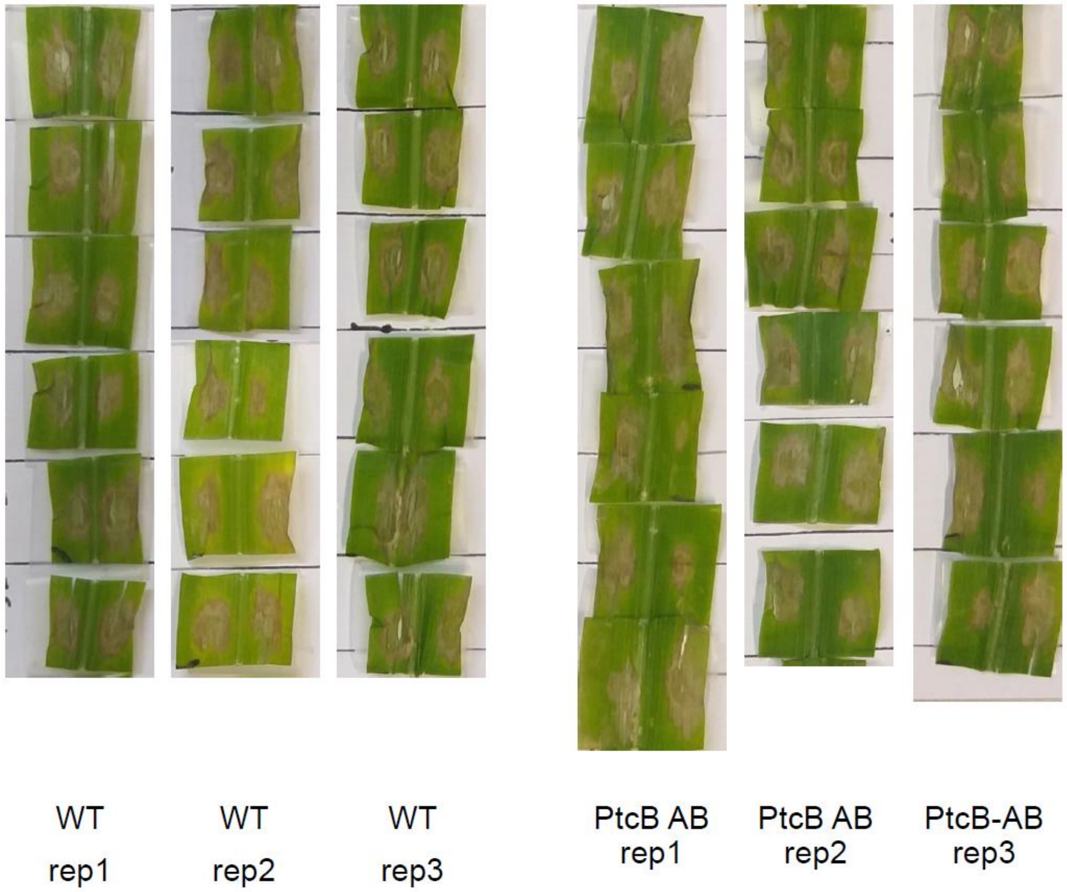

Figure S3. Quantitation of lesion areas from the virulence assays of PtcB AddBack and WT on maize leaves. Methods were as for Figure 9.

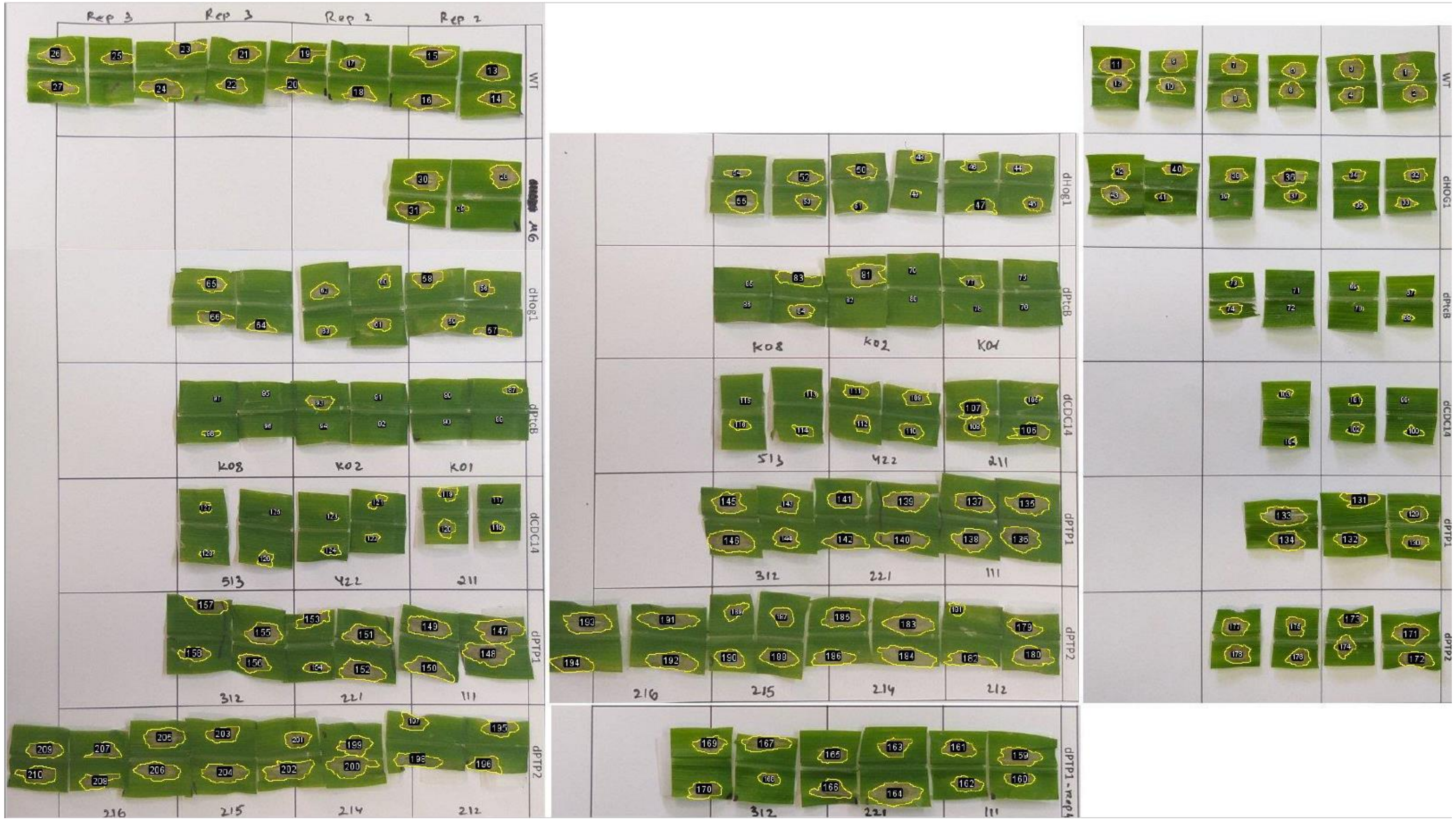

Figure S4. Quantitation of lesion areas from virulence assay of mutants and WT on maize leaves. Methods were as for Figure 9. Strain M6 (top left) is not related to this study.

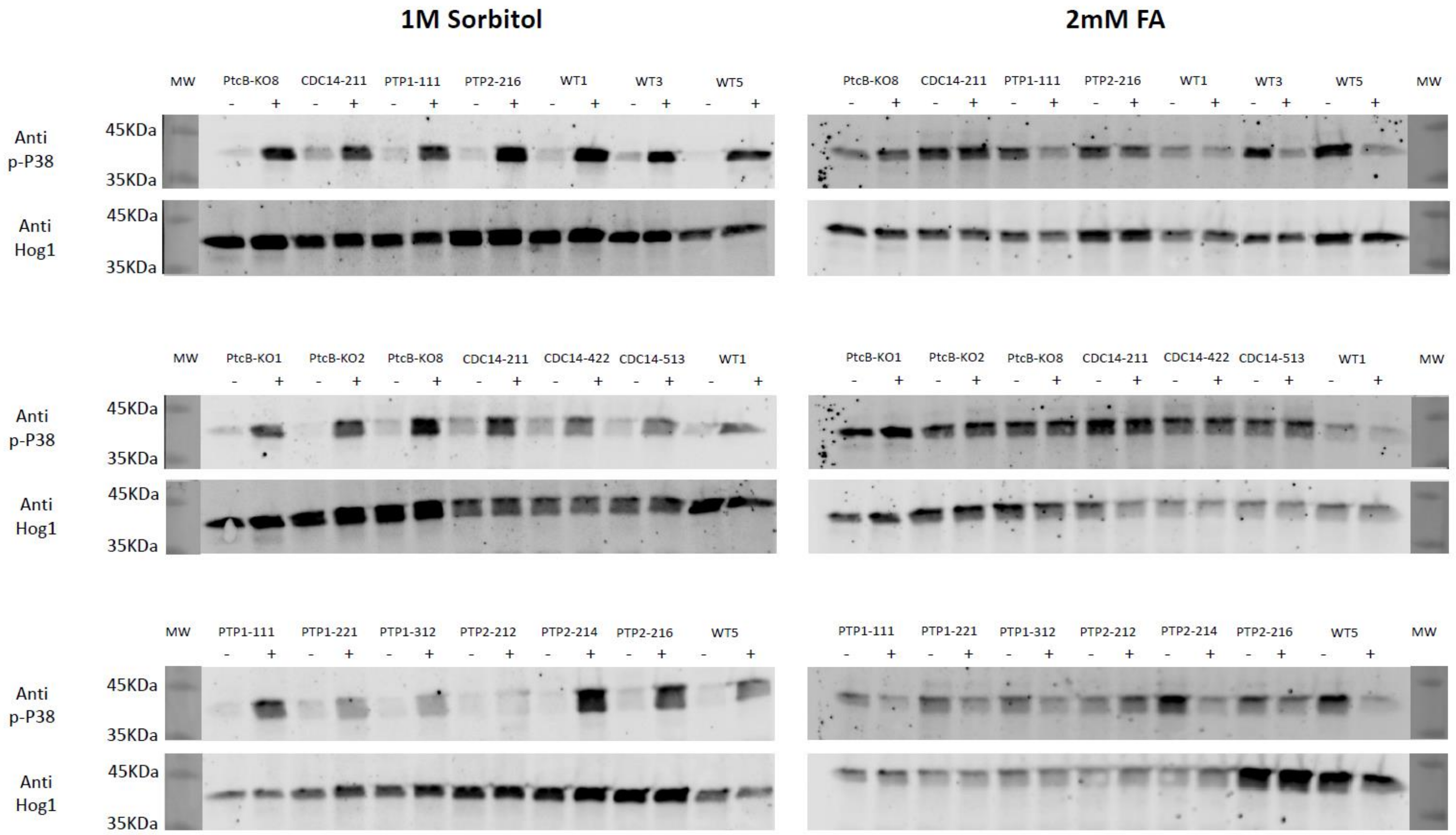

Figure S5. FA and sorbitol effect on Hog1 dual phosphorylation in WT and phosphatase mutants, in assayed by immunoblotting. All biological repeats for Figure 10: blots of total protein extract of WT and the different mutants, with (+) or without (−) sorbitol or FA induction.
